# A Novel Bifunctional Fusion Protein, Vunakizumab-IL22, for Protection Against Pulmonary Immune Injury Caused by Influenza Virus

**DOI:** 10.3389/fimmu.2021.727941

**Published:** 2021-08-24

**Authors:** Lei Han, Chenchen Shi, Xian Zeng, Lifeng Cen, Xiaobin Mei, Jiajun Fan, Dianwen Ju, Haiyan Zhu

**Affiliations:** ^1^Department of Biological Medicines & Shanghai Engineering Research Center of Immunotherapeutics, Fudan University School of Pharmacy, Shanghai, China; ^2^Division of Spine, Department of Orthopedics, Tongji Hospital, School of Medicine, Tongji University, Shanghai, China; ^3^Department of Nephrology, Changhai Hospital, Second Military Medical University, Shanghai, China

**Keywords:** bifunctional fusion protein, tissue repair, anti-inflammatory effects, H1N1 influenza A virus, IL-17A, IL-22, lung injury

## Abstract

Influenza A virus infection is usually associated with acute lung injury, which is typically characterized by tracheal mucosal barrier damage and an interleukin 17A (IL-17A)-mediated inflammatory response in lung tissues. Although targeting IL-17A has been proven to be beneficial for attenuating inflammation around lung cells, it still has a limited effect on pulmonary tissue recovery after influenza A virus infection. In this research, interleukin 22 (IL-22), a cytokine involved in the repair of the pulmonary mucosal barrier, was fused to the C-terminus of the anti-IL-17A antibody vunakizumab to endow the antibody with a tissue recovery function. The vunakizumab-IL22 (vmab-IL-22) fusion protein exhibits favorable stability and retains the biological activities of both the anti-IL-17A antibody and IL-22 *in vitro*. Mice infected with lethal H1N1 influenza A virus and treated with vmab-mIL22 showed attenuation of lung index scores and edema when compared to those of mice treated with saline or vmab or mIL22 alone. Our results also illustrate that vmab-mIL22 triggers the upregulation of MUC2 and ZO1, as well as the modulation of cytokines such as IL-1β, HMGB1 and IL-10, indicating the recovery of pulmonary goblet cells and the suppression of excessive inflammation in mice after influenza A virus infection. Moreover, transcriptome profiling analysis suggest the downregulation of fibrosis-related genes and signaling pathways, including genes related to focal adhesion, the inflammatory response pathway, the TGF-β signaling pathway and lung fibrosis upon vmab-mIL22 treatment, which indicates that the probable mechanism of vmab-mIL22 in ameliorating H1N1 influenza A-induced lung injury. Our results reveal that the bifunctional fusion protein vmab-mIL22 can trigger potent therapeutic effects in H1N1-infected mice by enhancing lung tissue recovery and inhibiting pulmonary inflammation, which highlights a potential approach for treating influenza A virus infection by targeting IL-17A and IL-22 simultaneously.

## Introduction

H1N1 influenza A virus, one of the major subtypes disseminated in humans, has been reported to cause acute respiratory illness such as acute lung injury, especially in patients with severe form. Lung function impairment caused by an excessive inflammatory response was reported to be the main cause of the deaths (1–4). Although a series of agents, such as neuraminidase inhibitors, have been used to improve the prognosis of patients, viral infection-induced tissue injury has been limitedly controlled, which finally result in high mortality in H1N1 influenza A virus-infected patients. Thus, novel approaches are urgently required for the treatment of H1N1 influenza A virus infection.

Interleukin 17A (IL-17A), a member of the IL-17 family, has been proven to play crucial roles in not only autoimmune disorders but also viral infectious diseases (5–10). During the infection process, IL-17A is secreted by a variety of immune cells, such as T helper (Th) cells, macrophages, and natural killer (NK) cells. Although appropriate levels of IL-17A can trigger the expression of a series of cytokines, chemokines, and antimicrobial peptides, such as IL-6, granulocyte-colony-stimulating factor (G-CSF), tumor necrosis factor α (TNF-α) and CXCL1, to kill pathogens, excessive IL-17A production results in severe inflammation and the progression of the disease. Recent studies showed that IL-17A was overexpressed in lung tissues after mice were infected with influenza A/PR/8 for 2 days, which caused the excessive recruitment and accumulation of inflammatory cells and serious lung injury (11). In addition, knockout of IL-17A gene could impair the production of IL-1β, IL-23 and TNF-α, which remarkably rescued pulmonary function and increased survival in virus-infected mice by attenuating inflammation, indicating that targeting IL-17A might be a potential approach for ameliorating the abnormal immune response during virus-induced lung injury (8, 12, 13). Indeed, neutralizing antibodies targeting IL-17A have been proven to be beneficial for treating influenza virus-induced tissue pathology in several preliminary investigations. Although the blockade of IL-17A signaling alleviates tissue damage and inflammation during virus infection in murine models, targeting IL-17A alone has limited efficacy in repairing the tissue damage caused by influenza A virus infection (14).

IL-22 plays a protective and regenerative role during inflammation (15). It does not directly regulate immune cells but targets barrier cells, such as the respiratory epithelium (16–19). Several studies have shown that IL-22 is a key factor in mucosal barrier defense and tissue repair (20–24). During influenza infection, IL-22 plays a key role in maintaining the epithelial integrity of the lung (25, 26). It has been proved to reduce lung inflammation caused by influenza A virus infection and protect against secondary bacterial infection by regenerating respiratory epithelial cells and enhancing tight junctions (27, 28). In light of these findings, it would be of interest to study whether simultaneously targeting IL-22 and IL-17A could be a potential approach for virus infection therapy.

In this study, a novel bifunctional fusion protein, vunakizumab-IL22 (vmab-mIL22), which has the activities of both IL-22 and the anti-IL-17A antibody, was generated as a novel potential approach for the treatment of influenza A virus infection. Two functional parts of vmab-mIL22 collaboratively delivered desired pharmacological outcome on influenza A virus infection models. The IL-22 payload promotes tissue repair, and the antibody part reduces IL-17A-mediated inflammatory response as well as enhances the tissue repair function of IL-22. This study demonstrated that vmab-mIL22 is a potential therapy for viral pneumonia, providing new insights for the development of biological drugs for the treatment of severe influenza.

## Materials and Methods

### Mice, Viruses, Infections, and Treatment of Virus-Infected Mice

All animal experiments were approved and performed in compliance with the Animal Ethics Committee of the School of Pharmacy, Fudan University. A/FM/1/47 (H1N1) was supplied by the Shanghai Centers for Disease Control & Prevention (Shanghai, China).

Male BALB/c mice aged 4-6 weeks, purchased from the Slaccas Company (Shanghai) were divided into six groups (n=7 for each group): Normal control (PBS); H1N1 + vehicle (PBS); H1N1 + 8.58 mg/kg vmab; H1N1 + 5 mg/kg mIL22Fc; H1N1 + 10.66 mg/kg vmab-mIL22; and H1N1 + 8.58 mg/kg vmab + 5 mg/kg mIL22Fc (combination); the dose of vmab and vmab-mIL22 are equimolar to that of mIL22Fc. Except for the normal group, the other mice were intranasally inoculated with H1N1 virals at a dose of 2 × LD50. Virus infection was carried out in the biosafety cabinet. Briefly, in a biosafety cabinet, the mice were anesthetized with isoflurane, and 15ul virus viral particle suspension was dropped on the left and right noses of the mice, respectively. The residual virus solution and instruments contaminated by the virus solution were subjected to high-pressure sterilization after the infection. The treatments were initiated 2.5 hours after intravenous injection of the virus. On the 5th day after infection, the mice were sacrificed, lung index (lung weight to body weight ratio) and histological examination were measured. The lung tissue was fixed in 4% formaldehyde buffer for histological examination.

### Cell Lines

ExpiCHO-S cells (Gibco, Thermo Scientific Ltd.) were cultured in ExpiCHO expression medium at 37°C, 8% CO_2_ with 120rpm shaking in an Erlenmeyer flask. HT29 (colorectal adenocarcinoma cells) and HepG2 (liver hepatocellular carcinoma cells) cells were cultured in Dulbecco’s modified Eagle’s medium supplemented with 10% fetal bovine serum at 37°C, 5% CO_2_. The culture medium and fetal bovine serum were purchased from GIBCO, Thermo Fisher Scientific Inc.

### Gene Synthesis, Cloning and Expression of vmab-mIL22, vmab and mIL22Fc

DNA inserts encoding the heavy-chain variable regions of the mouse IL-17A antibody (vunakizumab, vmab) were synthesized and cloned into a pTT5 expression vector containing the sequence for a human IgG_4_ heavy-chain constant region. DNA inserts encoding the light-chain variable regions of vmab were synthesized and cloned into a pTT5 expression vector containing the sequence for a human κ–light-chain constant region. For construction of the IL-17A antibody-mouse IL22 fusion protein (vmab-mIL22), the mouse IL-22 sequence along with a flexible (Gly4Ser)3 spacer was inserted at the C terminus of the vmab heavy chain using a polymerase chain reaction assembly procedure, resulting in the plasmid pTT5-vmab-mIL22. Genes coding for a mouse IL22 IgG_4_Fc fragment fusion protein carrying appropriate restriction sites at the 5’ and 3’ ends were synthesized, digested with restriction enzymes and cloned into the pTT5 expression vector, resulting in the plasmid pTT5-mIL22Fc. The corresponding antibody and fusion proteins were produced by liposome-mediated transient gene expression in ExpiCHO cells (Thermo Fisher Scientific) according to the vendor’s protocol. Two weeks after transfection, cell supernatants were harvested and purified over a Protein A affinity chromatography column (GE Healthcare) followed by size-exclusion chromatography (Superdex200 column 10/300 GL; GE Healthcare).

### SDS-PAGE

SDS-PAGE analysis was performed on 4-20% Tris-glycine gradient gels under reducing and non-reducing conditions. Each well contained 7 mg protein samples, and the gel was stained with Coomassie brilliant blue (Invitrogen) and decolorized by decolorizing solution (containing 30% methanol and 10% acetic acid).

### SEC-HPLC for Stability Analysis

The degree of aggregation and degradation of 5 mg/mL vmab-mIL22, vmab and mIL22Fc in PBS buffer at 37°C for 10 days was studied by SEC-HPLC. SEC analysis was performed using a HPLC system with a photodiode array detector (Waters Corporation, Mil-ford, MA). Fifty micrograms of protein were injected onto an XBridge BEH 200A SEC column (Waters) using PBS as the mobile phase, with a flow rate of 0.5 mL/min. The total run time was 30 min, and chromatographic separation was monitored *via* UV detection at 280 nm.

### Thermal Stability Analysis

For antibody molecules, each unfolding domain has a measurable transition temperature (Tm1-Tm2). Increasing Tm and Tagg values reflect an increase in conformational stability (29–32).

Tm and Tagg values were determined noninvasively in multicuvette arrays with the Unit^®^ device (UNCHAINED LABs, USA) by measuring the intrinsic fluorescence evoked by the intrinsic tryptophan residues. A temperature ramp with 0.3°C/min steps from 20°C to 95°C was performed. The concentration of the sample to be tested was 5 mg/ml, and the sample loading volume was 9 μL. The obtained raw data were fitted to a sigmoidal shape, and the inflection point of the fit was assigned as the melting point.

### Surface Plasmon Resonance (SPR)

The interaction of vmab-mIL22 or vmab with human or mouse IL17A was determined with an SPR assay performed on a Biacore T200 system (Biacore, GE Healthcare). Briefly, a human IgG capture antibody (Biacore, GE Healthcare) was preimmobilized on a CM5 sensor chip (GE Healthcare), and test antibodies were captured by flowing them through the chip. The final capture amount for the test antibodies was adjusted to an equal amount of 100 response units (RU) by adjusting the capture time. Then, human or mouse IL17A antigen was allowed to flow through the chip for 180 s followed by 800 s for dissociation. IL17A was tested at concentrations of 0.01, 0.02, 0.05, 0.1 and 0.2 μg/ml. The data were collected, and the affinities between vmab-mIL22 or vmab and IL17A were analyzed using Biacore Evaluation Software.

### *In Vitro* Bioactivity Assay of vmab-mIL22

IL-17A stimulated HT29 cells to produce and release Groα into the cell supernatant. In order to evaluate the biological activity of vmab-mIL22 in neutralizing IL-17A, HT29 cells were stimulated with 5nM IL-17A in the presence of different concentrations (ranging from 100 nM to 15.24 pM) of vmab-mIL22. In 96 well plates, there were about 20,000 living cells in each flat bottom well and cultured at 37°C and 5% CO_2_ for 48 hours. Groα in the supernatant was determined by ELISA (R&D). The ELISA was performed according to the manufacturer’s instructions. Vmab was used as positive control to block the function of IL-17A. The mean values of repeated wells were plotted and the standard deviation was calculated using the four parameter dose response inhibition function in Graphpad prism.

### *In Vitro* Bioactivity Assay of IL-22

mIL-22 induces phosphorylation of STAT3 in HepG2 cells. To assess the bioactivity of mIL22 in inducing STAT3 phosphorylation, HepG2 cells were cultured in serum-free modified Eagle’s medium in a 6-well plate (5×10^5^ cells per well) at 37°C and 5% CO_2_ for 12 hours. Subsequently, a 10 nM concentration of the protein to be investigated was added. After incubation at 37°C for 30 minutes or 1.5 h, the cells were washed with PBS and lysed with 20 μL of RIPA buffer for 5 minutes. The supernatant of cell lysate was used for SDS-PAGE after centrifugation at 10000 rpm for 10 minutes. The mouse anti-human phospho-STAT3 (Biolegend) and goat anti-mouse HRP (Merck) were used for Western blot analysis. The immunoblotting signals were visualized using a Western Luminescent Detection kit (Thermo Fisher Scientific).

### Histopathology Study

The lung tissue was immediately fixed with 10% neutral formalin PBS buffer, embedded in paraffin, and cut into 5-7 μm section. The sections were dewaxed and stained with hematoxylin eosin (H&E) or periodate Schiff (PAS) to observe the histological changes and the number of goblet cells. The images were obtained by microscope with magnification of 100 or 200 times (panoramic MIDI, 3dhistech).

### Immunohistochemistry and Cytokine Detection

Immunohistochemical analysis was performed using antibodies against MUC2 and ZO1 (Abcam) according to the manufacturer’s instructions. The lung homogenates were prepared in PBS at a concentration of 100 mg tissue/mL. BCA Kit (Thermo Fisher Scientific) was used to detect the total protein concentration in the homogenates. ELISA kits were used to detect the following cytokines in lung tissue: IL-1β (eBioscience), IL-10 (eBioscience) according to the manufacturer’s instructions and mouse serum was used for HMGB1 (eBioscience) detection. The absorbance was measured at 450 nm wavelength using a Multi-Detection Microplate Reader (BioTek).

### RNA Sequencing and Bioinformatics Analysis

Total RNA was isolated from lung tissue samples and followed by RNA integrity, purity and concentration evaluations. Concentrated mRNAs were randomly broken into short fragments to generate double-stranded (ds) cDNAs. Poly A tails and sequencing connectors were added to cDNA end sequences. ds-DNAs were digested into single-stranded (ss) DNAs by the USER enzyme, and PCR amplification was conducted for 15 cycles to establish the final cDNA library. Finally, sequencing was performed using 2*151 sequencing mode on Illumina sequencer. RNA sequencing raw data was subjected to data quality control using fastqc algorithm, qualified sequence segments were aligned to mouse reference genome (mm10) using STAR software (version 2.7.1a) (33). Gene read counts were calculated using featureCounts algorithm in Rsubread R package (34). Differential gene expression (DEG) analysis was conducted using edgeR R package and Benjamini and Hochberg (BH) method was used to calculate false discovery rate adjusted p values (35). Volcano plot was drawn using EnhancedVolcano R package to show the DEG results. Gene expression TPM values were calculated using kallisto algorithm (v0.46.1) and transformed by Z-score for heatmap visualization (Complexheatmap R package) (36, 37). All bioinformatics analysis was performed using R software (version 4.0.3) except that gene ontology and pathway enrichment analysis were performed using Enrichr website (https://maayanlab.cloud/Enrichr/) (38). Specifically, up-regulated and down-regulated DEG gene lists were submitted to EnrichR website for KEGG pathway enrichment analysis. Pathways with FDR-adjusted p value <= 0.01 were selected as significantly over-represented pathways and FDR-adjusted p values were -log10 transformed to present the results in bar charts.

### Statistical Analysis

Values were expressed as mean ± SEM. Multiple group comparisons were performed using one-way analysis of variance (ANOVA) followed by Bonferroni *post hoc* test using the software of SPSS. p-value of less than 0.05 (p < 0.05) was considered to be significant. Statistically significant differences are indicated as *P < 0.05, **P < 0.01 or ***P < 0.001.

## Results

### Production and *in Vitro* Characterization of a Fusion Protein of the IL17A Antibody and IL22 (vmab-mIL22)

**Figure 1:**
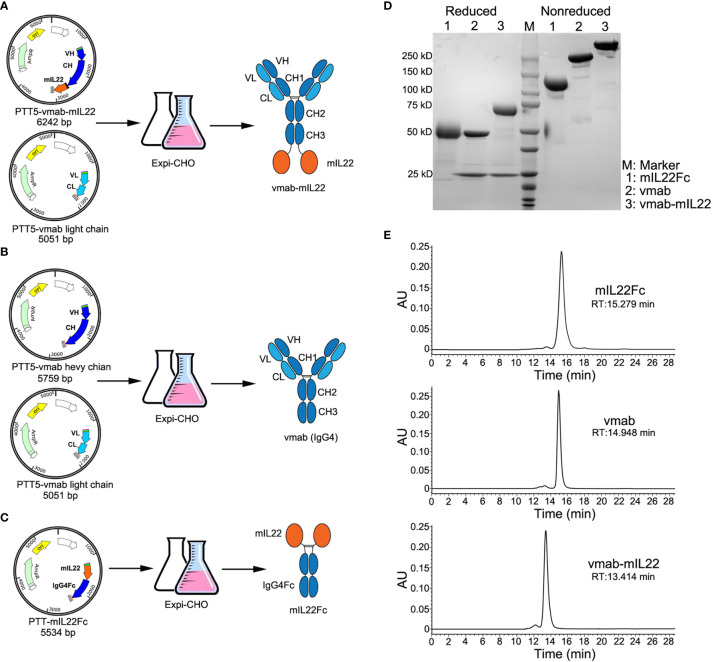
Cloning, expression, and characterization of vmab-mIL22, vmab and mIL22Fc. **(A)** Schematic representation of the cloning strategy and expression data for vmab-mIL22. Mouse IL22 fused to the C-terminus of the heavy chain of vunakizumab (vmab) *via* a 15-amino acid (aa) linker. **(B)** Schematic representation of the cloning strategy and expression data for vmab. **(C)** Schematic representation of the cloning strategy and expression data for mIL22Fc (mouse IL22 and IgG4 Fc fusion protein). **(D)** SDS-PAGE analysis of mIL22Fc (lane 1), vmab (lane 2) and vmab-mIL22 (lane 3) under either reducing or nonreducing conditions. **(E)** SEC-HPLC analysis of mIL22Fc, vmab and vmab-mIL22. VH: antibody variable heavy chain. VL: antibody variable light chain. CH: antibody heavy chain constant region. CL: antibody light chain constant region. mIL22: mouse IL-22. RT: retention time.

vmab-mIL22 was constructed by genetically fusing mouse IL22 (mIL22) to the C-terminus of the heavy chain of an anti-IL-17A antibody (vunakizumab, vmab) *via* a (Gly4Ser)3 linker. The DNA sequence coding for the fusion proteins were constructed into the mammalian expression vector pTT5, yielding the plasmid pTT5-vmab-mIL22. The plasmid together with the pTT5-vmab-light chain, which contains the gene encoding the light chain of vunakizumab, was used in the production of the vmab-mIL22 fusion protein (**Figure 1A**).

Using a similar method, vmab and mouse IL22Fc fusion protein (mIL22Fc) was prepared as a control (**Figures 1B, C**). These proteins were purified by protein A affinity chromatography, and further purified by size exclusion chromatography. The purity of the collected proteins was analyzed by SDS-PAGE and SEC-HPLC. As shown in **Figure 1D**, SDS-PAGE analysis of vmab-mIL22 showed bands of the expected size, with a heavy chain larger than that of the parental antibody vmab and a high purity on SDS/PAGE and SEC-HPLC (**Figures 1D, E**).

**Figure 2:**
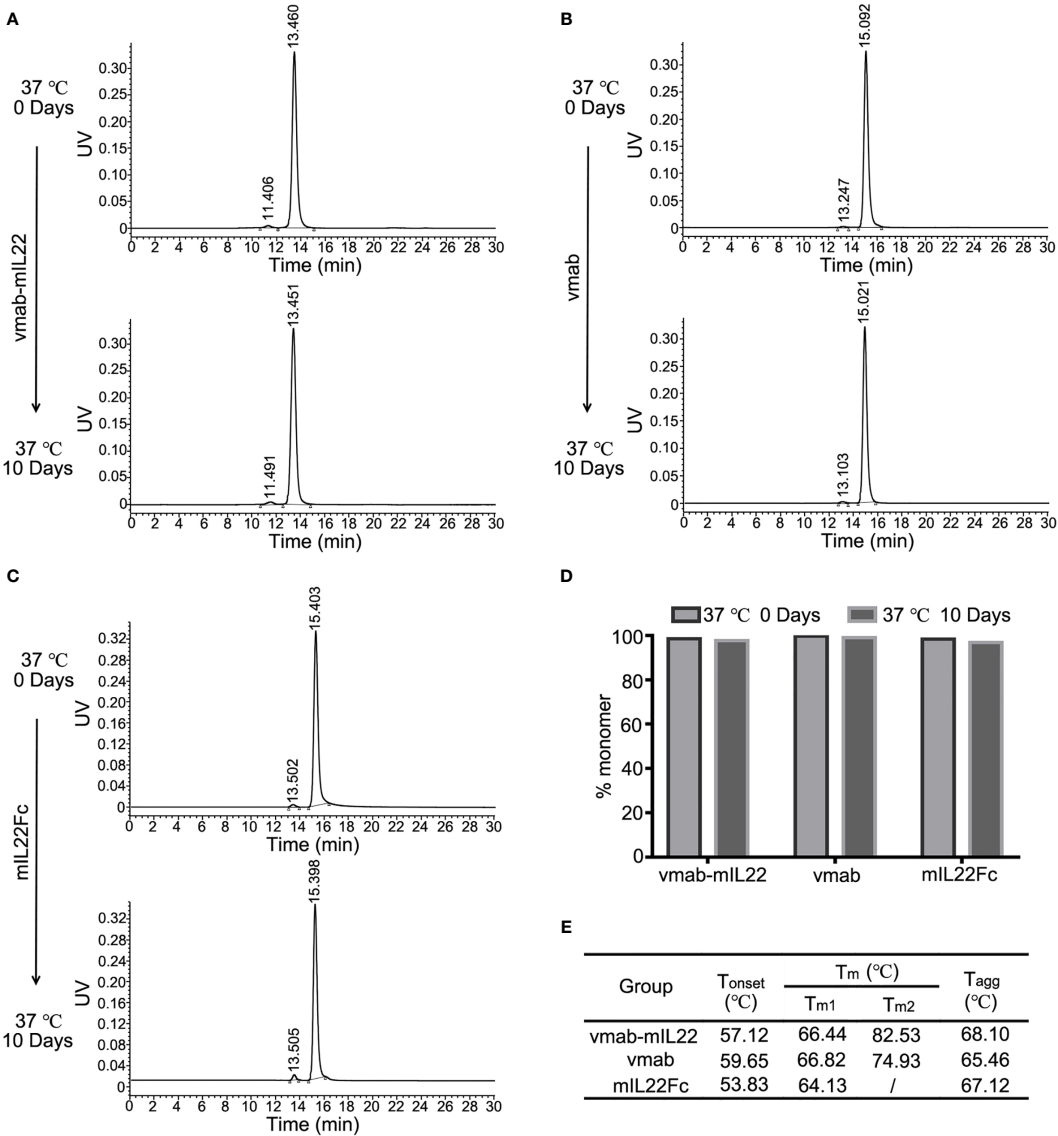
Stability analysis of mIL22-Fc, vmab and vmab-mIL22 at 37°C for 10 days. HPLC-SEC profiles of **(A)** vmab-mIL22, **(B)** vmab and **(C)** mIL22Fc at 0°C or 37°C for 10 days. **(D)** Compared with that observed at the 0 day timepoint, the measurable aggregation (agg) of mIL22-Fc, vmab and vmab-mIL22 did not increase evidently or show measurable degradation after 10 days at 37°C (clip). **(E)** Thermal stability analysis of vmab-mIL22, vmab and mIL22-Fc using intrinsic protein fluorescence measurements. The final values were averages of three independent measurements. Increasing Tm and Tagg values reflect an increase in macroscopic conformational stability. Tonset (°C), protein unfolding temperature; Tm, melting temperature; Tagg, aggregation temperature.

The stability of vmab-mIL22 was determined afterwards. The degree of aggregation and degradation of 5 mg/mL vmab-mIL22 in PBS buffer at 37°C over 10 days was studied by SEC-HPLC (**Figure 2A**). For vmab-mIL22 at 37°C for 10 days, only 2.35% of polymer was detected **(** **Figure 2A**), and there were no degradation fragments, similar to vmab (**Figure 2B**). The main peak component of vmab-mIL22 decreased from 98.57% to 97.64%, and the main peak component of vmab decreased from 99.69% to 99.03% (**Figure 2D**). Although the degree of aggregation of mIL22Fc increased slightly, the main peak component was still more than 95% (**Figures 2C, D**). Compared to that of vmab, the molecular integrity of vmab-mIL22 was not evidently changed, which indicates that vmab-mIL22 exhibits favorable stability *in vitro*.

We also measured the thermal stability of vmab-mIL22 using intrinsic protein fluorescence measurements. Tm and Tagg values were measured using the Optim^®^2 system. All studied proteins were diluted to an assay concentration of 5 mg/mL using 25 mM Tris buffer (3 mM KCl, 140 mM NaCl, pH 7.4). As shown in **Figure 2E**, vmab-mIL22 and vmab showed comparable Tm1, while vmab-mIL22 had higher Tm2 and Tagg than vmab. These results suggested that the structure of vmab-mIL22 was more stable than that of vmab.

### vmab-mIL22 Exhibits Dual Functionality, and the Functional Integrity of the Cytokine Payload Is Preserved

**Figure 3:**
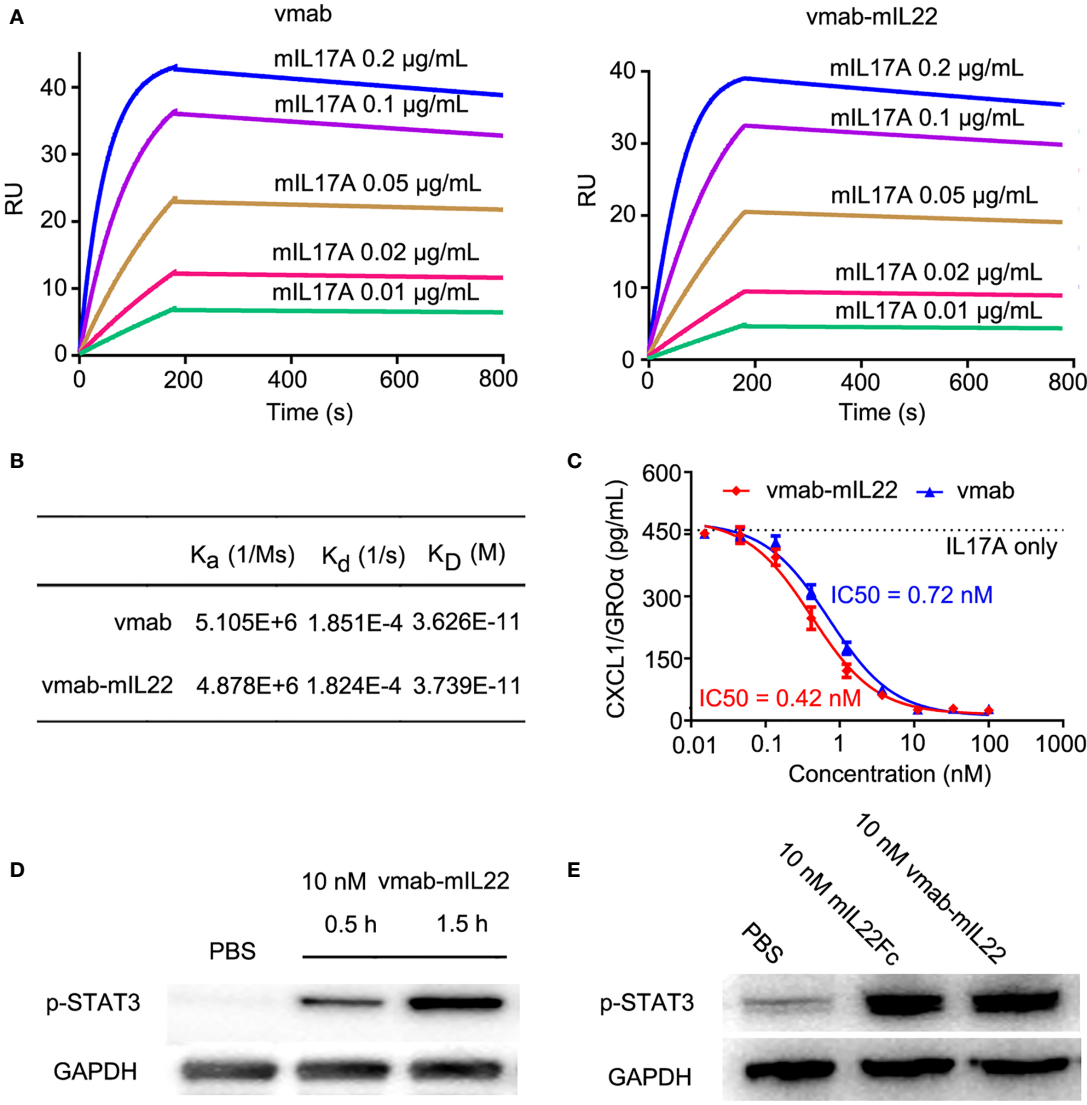
vmab-mIL22 exhibits dual functionality, and the functional integrity of the cytokine payloads is preserved. **(A, B)** Biacore sensorgrams of the interaction of vmab with mIL17A and BIAcore sensorgrams of the interaction of vmab-mIL22 with mIL17A. vmab-mIL22 retained a high affinity for the cognate antigen, similar to that of the parental antibody Vmab, which was confirmed by surface plasmon resonance analysis. Sensorgrams display the response values at IL17A concentrations of 0.01 to 0.2 μg/mL, with a CM5 chip and capture with 100 RU vmab or vmab-mIL22. The equilibrium dissociation constant (K_D_) was calculated using k_d_/k_a_ for each measurement. k_d_, dissociation rate constant or off-rate constant; k_a_, association rate constant or on-rate constant. Binding was biphasic, and the data were fit with heterogeneous ligand models. **(C)** *In vitro* inhibition of functional activity by vmab-mIL22. Inhibition of Groα release from HT-29 cells as a measure of IL-17A inhibition. HT29 cells were stimulated with IL-17A (5 nM) in the presence of various concentrations of vmab-mIL22. vmab was used as a positive control. Groα levels in the supernatants were measured by ELISA from R&D systems. **(D)** Western blot analysis of STAT3 phosphorylation (p-STAT3) induced by 10 nM vmab-hIL22 in HepG2 cells at different times. **(E)** Western blot analysis of STAT3 phosphorylation (p-STAT3) induced by 10 nM mIL-22Fc or vmab-mIL22 in HepG2 cells at 1.5 h Serum-starved cells (3×10^6^/mL) were stimulated with PBS, 10 nM mIL22-Fc or Vmab-mIL22 for 1.5 h at 37°C. Whole-cell lysates were resolved on denaturing gels, transferred, and immunoblotted with a polyclonal anti-phospho-STAT3 antibody.

To assess the binding affinity and kinetic parameters of vmab-mIL22 with mouse IL-17A (mIL17A), SPR analysis was conducted with vmab-mIL22 captured as the ligand and different concentrations of mIL-17A flowed as the analyte. IL-17A antibody vmab was taken as a positive binding control. As shown in **Figures 3A, B**, vmab-mIL22 had a similar affinity as vmab for mIL-17A. These data indicated that the presence of mIL22 on the C-terminus of the heavy chain of vmab had no effect on the binding of the antibody to mIL-17A.

To determine the activity of vmab-mIL22 against human IL-17A, HT-29 cells were stimulated with 5 nM IL-17A in the presence of several different concentrations of vmab-mIL22. As shown in **Figure 3C**, vmab-mIL22 inhibited the IL-17A-induced secretion of GRO-α in a dose-dependent manner and blocked IL-17A in a manner similar to vmab. The IC50 values of vmab-mIL22 and vmab were 0.4195 nM and 0.7163 nM, respectively. The above data indicated that the presence of IL-22 on the vmab backbone did not affect the inhibition of IL-17A activity by the antibody portion of vmab-mIL22.

The functional integrity of the IL22 payload was verified by Western blot analysis of mIL22-induced STAT3 phosphorylation in HepG2 liver hepatocellular carcinoma cells. As shown in **Figure 3D**, vmab-mIL22 induced upregulation of pY705-STAT3 levels in a time-dependent manner. We also tested the expression of pY705-STAT3 after 1.5 h of mIL22Fc and vmab-mIL22 treatment in the same cells. As shown in **Figure 3E**, the expression of pY705-STAT3 was upregulated 1.5 h after mIL22Fc and vmab-mIL22 treatment. These results indicated that the functional integrity of the cytokine payload is preserved, and vmab-mIL22 retained biological cytokine activity comparable to that of mIL22.

In summary, our results demonstrated that vmab-mIL22 retained the biological activity of the antibody and IL-22.

### Compared to vmab and mIL22 Alone, vmab-mIL22 Exhibited a Superior Ability to Control H1N1-Induced Lung Immune Injury in Mice

The therapeutic efficacy of vmab-mIL22 was studied in mice with influenza A virus (H1N1)-induced lung injury. We first investigated the effects of different doses of vmab-mIL22 on pulmonary edema caused by H1N1 infection at 5 days post-inoculation. vmab-mIL22 significantly improved pulmonary edema caused by H1N1 infection in a dose-dependent manner (p <0.01) (data not shown).

**Figure 4:**
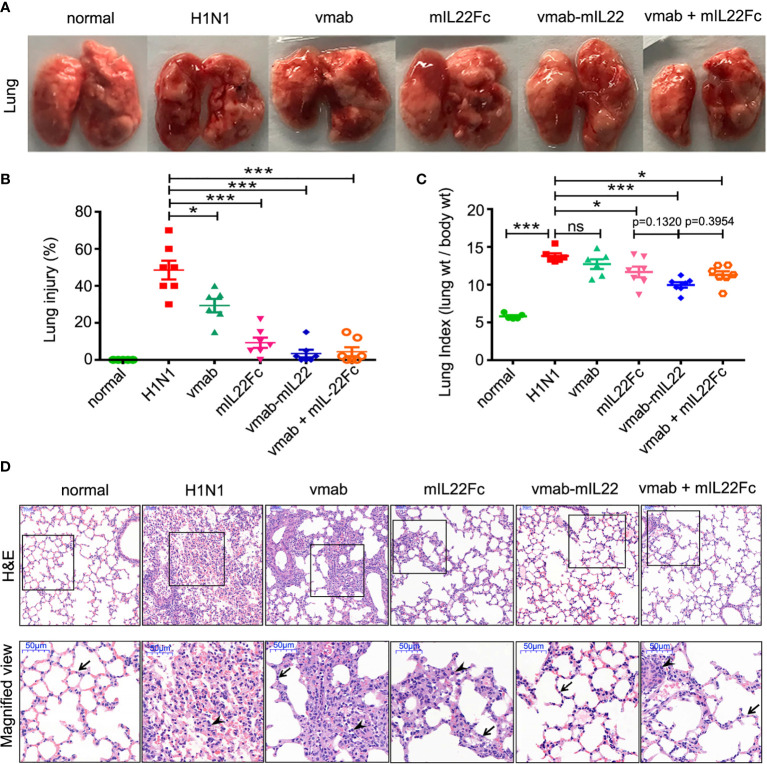
The therapeutic efficacy of vmab-mIL22 against H1N1-induced lung immune injury. **(A)** Representative pulmonary edema showing the pathology of the lung. **(B, C)** Assessment of the therapeutic effect of vmab-mIL22 on pulmonary edema and lung index scores H1N1 infection, compared with saline, vmab, mIL22Fc, the combination of vmab and mIL22Fc and healthy mice. Mice were randomly divided into six groups. There are seven mice in each group (n = 7). Values were expressed as mean ± SEM. Multiple group comparisons were performed using one-way analysis of variance (ANOVA) followed by Bonferroni *post hoc* test using the software of SPSS. p-value of less than 0.05 was considered to be significant. **(D)** Representative histological sections of the lung tissues from H1N1 virus-infected mice receiving each therapy or saline treatment were stained with hematoxylin and eosin. Bars, 50 μm. Wedge indicates accumulation of mononuclear cells, and black arrow indicates the alveolar wall. *P < 0.05 or ***P < 0.001. NS, not significant.

Next, to verify the synergistic therapeutic effect of the dual functional protein vmab-mIL22, the effects of vmab or mIL22Fc alone or vmab and mIL22Fc in combination on pulmonary edema caused by H1N1 infection were investigated. Compared to that in vmab or/and mIL22Fc-treated mice, infection-induced lung edema was significantly more attenuated in vmab-mIL22-treated mice (p < 0.001) (**Figures 4A–C**). At 6 days post-inoculation, H1N1 virus-infected lungs displayed highly edematous, congestive, and hemorrhagic changes, which were improved by vmab-mIL22 administration (**Figure 4A**). The pathology of the lung was determined after H1N1 infection and each therapy by hematoxylin-eosin staining (**Figure 4D**). Consistent with the degree of pulmonary edema, severe pathological injury was observed in the nontreated infected group on day 5 post-inoculation, including frequent cases of hyperemia, alveolar and bronchial structural damage. Neutrophils infiltration, as indicated by the cells with lobulated nuclei could also been observed in virus-infected lung tissues. In comparison, lungs from mice treated with vmab-mIL22, vmab or mIL22 showed fewer histologic changes, fewer infiltrated leukocytes and less pathological damage. Indeed, vmab-mIL22 exhibited more potent function of attenuating virus-induced lung injury than vmab and mIL22 (**Figures 4C, D** **)**. These results demonstrated that vmab-mIL22 could effectively ameliorate the severity of disease after H1N1 virus infection. Vmab-mIL22 showed dual therapeutic effects of reducing inflammation and repairing tissue damage.

**Figure 5:**
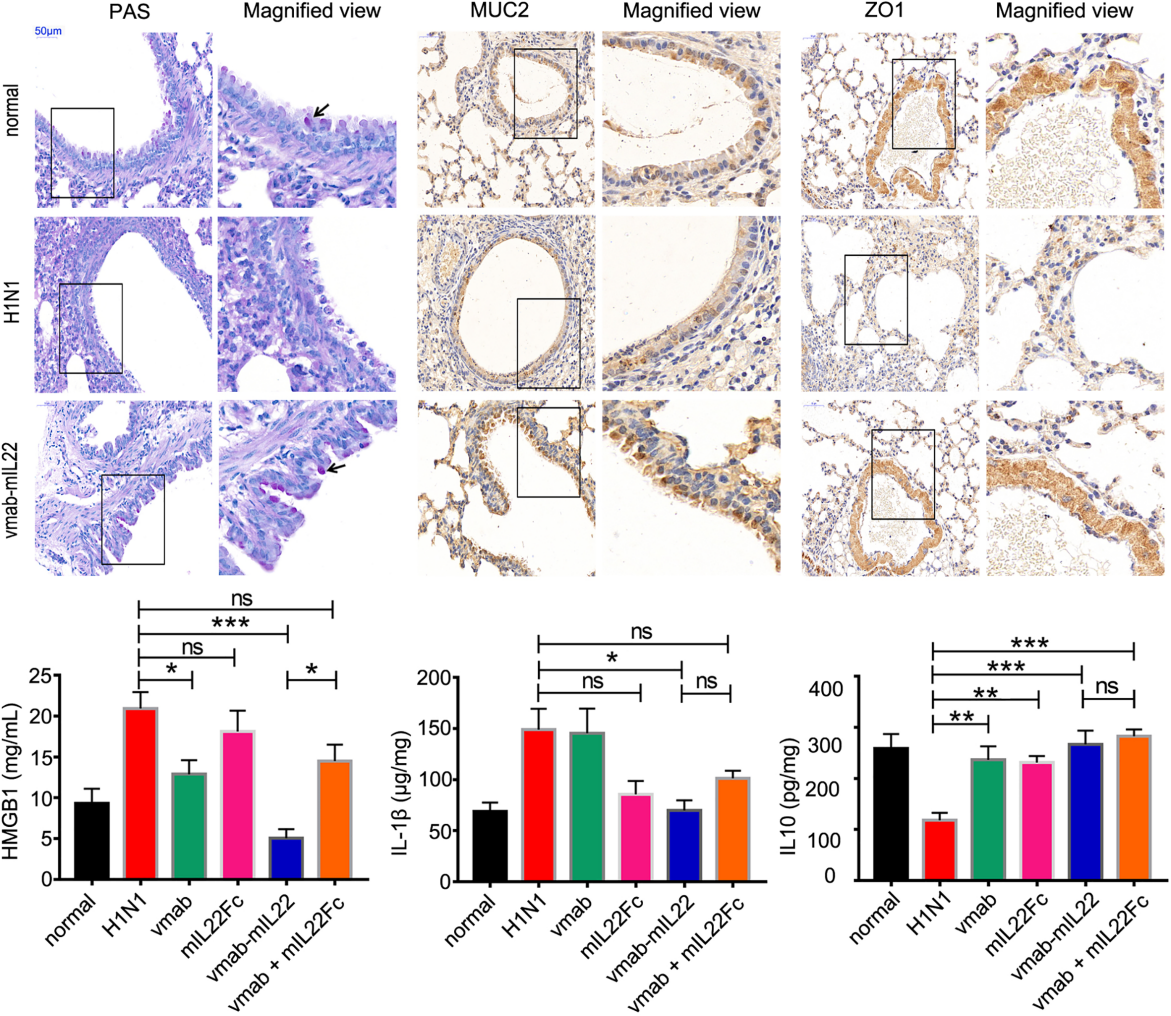
Vmab-mIL22 treatment increased epithelial repair and modulated inflammatory cytokine levels. **(A)** Recovery of pulmonary goblet cells and the mucus layer in the vmab-mIL22 treatment group. Pulmonary goblet cells are visualized by PAS staining. Black arrows indicate the goblet cells. MUC2 and ZO1 protein expression was assayed *via* immunohistochemistry. **(B)** Cytokines in the serum or lung homogenate were assayed using ELISA.There are seven mice in each group (n = 7). Values were expressed as mean ± SEM. Multiple group comparisons were performed using one-way analysis of variance (ANOVA) followed by Bonferroni *post hoc* test using the software of SPSS. p-value of less than 0.05 was considered to be significant. *P < 0.05, **P < 0.01 or ***P < 0.001. NS, not significant.

Goblet cells are the major secretory cells of acidic mucin glycoproteins, a major macromolecular component of the lung respiratory epithelium. To validate the effect of vmab-mIL22 on tissue damage repair, the goblet cell number and mucin expression were calculated in tissue sections by periodic acid Schiff (PAS) staining or immunohistochemistry against MUC2 and ZO1. As shown in **Figure 5A**, the goblet cell number and the expression of MUC2 and the tight junction protein ZO1 were reduced in H1N1-infected mice, indicating destruction of airway epithelial cell tight junctions and damage to the mucin layer structure, which were improved by vmab-mIL22 administration.

Interleukin-1β (IL-1β) is considered a central cytokine and accompanies early inflammation (39), and high mobility group box 1 (HMGB1) protein is a late mediator of inflammation that is released from IAV-infected epithelial cells, acting as a signal for inflammatory activation (40, 41). The actions of HMGB1 are also strongly associated with lung immunopathology (42, 43). As shown in **Figure 5B**, vmab-mIL22 treatment significantly reduced the levels of serum HMGB1 and pulmonary IL-1β, while the expression of interleukin 10 (IL-10) was upregulated, indicating that cell damage was attenuated and the inflammatory level was reduced. Importantly, vmab-mIL22 treatment exhibited better efficacy in reducing inflammatory molecule production than vmab or mIL22 cytokine alone or in combination.

These results indicate that vmab-mIL22 triggers the expression of MUC2 and ZO1, as well as the modulation of cytokines such as IL-1β, HMGB1 and IL-10, indicating the recovery of pulmonary goblet cells and the mucus layer and the suppression of excessive inflammation in mice after influenza A virus infection.

### A Weakened Inflammatory Response and Increased Tissue Repair Were Involved in the Transcriptomic Changes Resulting From vmab-mIL22 Treatment

**Figure 6:**
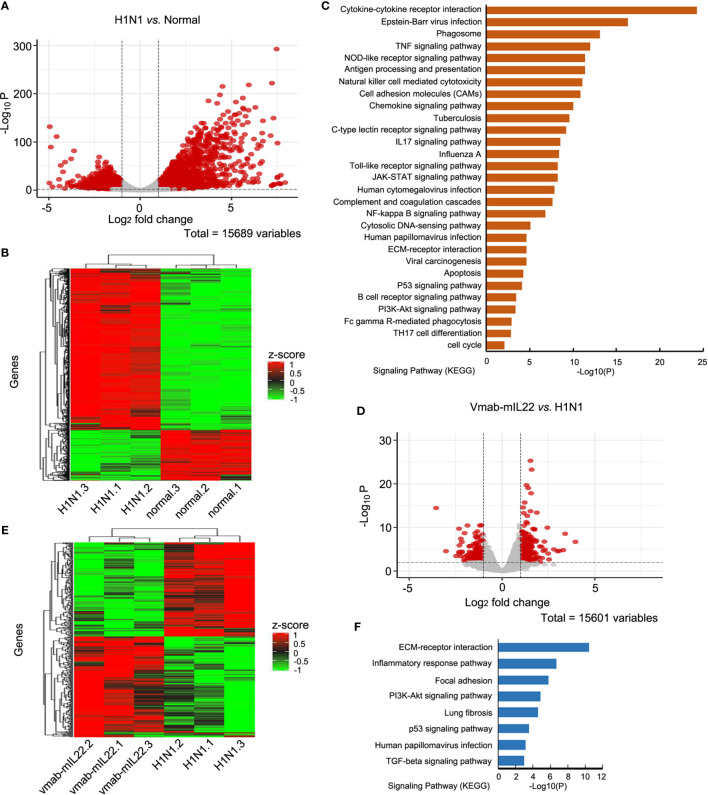
Transcriptomics changes of H1N1 infection and vmab-mIL22 treatment. **(A, B)** The differential expressed gene (DEG) analysis results were shown in volcano plots and differential expressed (DE) genes (|log2-fold change| >=1.0 and BH-adjusted P <= 0.05) between H1N1-infected and normal mice were selected for cluster analysis in heatmap. Among the DE genes, a set of 1823 genes showed upregulated expression, and the remaining 1340 genes showed downregulated expression. Each row represents an individual gene, and each column represents an individual sample. **(C)** Pathway analysis of genes found to be significantly upregulated in H1N1-infected mice. The top 29 significantly affected signaling pathways are shown. **(D, E)** The DE genes between vmab-mIL22-treated and H1N1-infected mice were selected for cluster analysis in heatmap. Among the DE genes, a set of 349 genes were upregulated, and the remaining 253 genes were downregulated. **(F)** Pathway analysis of genes found to be significantly downregulated in vmab-mIL22-treated mice. Eight significantly affected signaling pathway is shown.

To probe the underlying mechanisms of the therapeutic effect of vmab-mIL22, we analyzed the transcriptomic changes between lung tissues from noninfected control, H1N1-infected, and vmab-mIL22-treated mice. A total of 3163 genes were identified to be differentially expressed between H1N1-infected and noninfected control mice (fold change ≥ 2, p < 0.01). All detected genes were shown in volcano plot (**Figure 6A** **)** and those differentially expressed genes were visualized by heatmap (**Figure 6B**). Among these genes, 1823 were transcriptionally upregulated, and the remaining 1340 were negatively regulated. Gene Ontology enrichment analysis indicated that the differentially expressed genes mainly clustered into functional groups: cytokine-mediated signaling pathway (GO:0019221), inflammatory response (GO:0006954), cellular response to cytokine stimulus (GO:0071345), chemokine-mediated signaling pathway (GO:0070098), positive regulation of leukocyte chemotaxis (GO:0002690), positive regulation of cytokine production (GO:0001819), lymphocyte chemotaxis (GO:0048247), neutrophil migration (GO:1990266), and neutrophil-mediated immunity (GO:0002446) **(** **Figure 6C** **)**. In particular, the genes associated with immune and inflammatory responses were highly overexpressed among the upregulated genes, which indicated that the overabundant production of inflammatory responses is responsible for the symptoms and pathogenesis of lethal H1N1 virus infection.

Furthermore, genes involved in alveolar development and surface tension regulation were also differentially regulated. Epithelial genes surfactant protein A1 (Uteroglobin) (Sftpa1), surfactant proteins B (Sftpb) and C (Sftpc), and Secretoglobins (Scgb1a1, Scgb1c1, Scgb3a1, Scgb3a2) were downregulated in H1N1-infected mice *versus* control mice (**Table 1**). Collagen genes were upregulated, which is characteristic of pulmonary fibrosis (**Table 2**). The infection-induced changes of expression levels of these genes were significantly rescued in vmab-mIL22-treated mice, which indicated that vmab-mIL22 increased tissue repair and decreased collagen deposition. Moreover, pathway enrichment analysis indicated that the significant pathways of downregulated genes in the vmab-mIL22 treatment group mainly involved focal adhesion, the inflammatory response pathway, P53 signaling, the TGF-β signaling pathway, lung fibrosis, etc. (**Figures 6D–F** **)**, which indicated that vmab-mIL22 treatment may increase epithelial repair by attenuating the excessive inflammatory response and subsequent collagen deposition.

**Table 1:** Differentially expressed epithelial genes.

Gene Name	Protein Name	H1N1 *vs.* Nomal (Log2FC)	Treatment *vs.* H1N1 (Log2FC)	Protein Function (UniProt Accession)
Sftpa1	Pulmonary surfactant-associated protein A	-1.144	1.083	Lowers the surface tension in the alveoli of the lung; essential for normal respiration (P35242)
Sftpb	Pulmonary surfactant-associated protein B	-1.249	0.534	Promotes alveolar stability by lowering the surface tension (P50405)
Sftpc	Pulmonary surfactant-associated protein C	-2.055	1.333	Promotes alveolar stability by lowering the surface tension (P21841)
Scgb1a1	Uteroglobin	-1.556	1.201	Binds phosphatidylcholine, phosphatidylinositol and polychlorinated biphenyls (PCB) and weakly progesterone; potent inhibitor of phospholipase A2 (Q06318)
Scgb1c1	Secretoglobin family 1C member 1	-2.126	1.703	Q7M742
Scgb3a1	Secretoglobin family 3A member 1	-2.903	1.132	Secreted cytokine-like protein. Inhibits cell growth *in vitro* (Q920D7)
Scgb3a2	Secretoglobin family 3A member 2	-3.102	1.719	Strongly inhibits phospholipase A2 (PLA2G1B) activity. Seems to have anti-inflammatory effects in the respiratory epithelium. Also has anti-fibrotic activity in the lung (Q920H1)

Protein fuction was summarized from the UniProt database.

**Table 2:** Differentially expressed basement membrane genes.

Basement membrane genes	Protein Name	H1N1 *vs.* Nomal (Log2FC)	Treatment *vs.* H1N1 (Log2FC)	Protein Function (UniProt Accession)
Col1a1	Collagen alpha-1(I) chain	1.543	-1.198	Type I collagen is a member of the group I collagen (Fibril-forming collagen) (P11087)
Col1a2	Collagen alpha-2(I) chain	1.248	-1.038	Fibril-forming collagen (Q01149)
Col5a1	Collagen alpha-1(V) chain	1.033	-1.072	Fibril-forming collagen. It is a minor connective tissue component with nearly ubiquitous distribution (O88207)
Col5a2	Collagen alpha-2(V) chain	1.457	-1.302	Fibril-forming collagen. Type V collagen is a key determinant in the assembly of tissue-specific matrices (Q3U962)
Col5a3	Collagen type V alpha 3 chain	1.201	-1.581	Extracellular matrix structural constituent (Q9JLI2)
Col9a3	Collagen, type IX, alpha 3	1.878	/	Extracellular matrix structural constituent (A2ACT7)
Col3a1	Collagen alpha-1(III) chain	1.508	/	Involved in regulation of cortical development. (P08121)
Col11a2	Collagen alpha-2(XI) chain	1.034	/	Plays an important role in fibrillogenesis by controlling lateral growth of collagen II fibrils (Q64739)
Col12a1	Collagen alpha-1(XII) chain	/	-1.487	Cell adhesion; endodermal cell differentiation (Q60847)
Col14a1	Collagen alpha-1(XIV) chain	/	-1.120	Plays an adhesive role by integrating collagen bundles (Q80X19)
Col6a6	Collagen alpha-6(VI) chain	/	-1.215	Collagen VI acts as a cell-binding protein. (Q8C6K9)
Col28a1	Collagen alpha-1(XXVIII) chain	/	-1.753	May act as a cell-binding protein (Q2UY11)
Col3a1	Collagen alpha-1(III) chain	/	-1.037	Involved in regulation of cortical development. (P08121)

Protein function was summarized from the UniProt database.

## Discussion

Influenza remains a serious global health threat. Despite the vigorous promotion of vaccination strategy, there is little understanding of how to treat pneumonia caused by severe influenza in cases with ineffective vaccination (44). Due to the need to take these drugs early after the onset of the disease, and the emergence of drug-resistant virus strains, the therapeutic effect of direct anti-influenza drugs cannot be guaranteed (45–48). The host response to influenza virus infection has always been a research hotspot to determine whether various aspects of this response can be used for the treatment. In this process, cytokines play an important role in regulating the protective and repair processes, and may also cause tissue damage.

IL-17A and IL-22 are well-characterized critical cytokines in the rapid response to infectious agents, and the functions of both cytokines partially overlap and are in part complementary. IL-22 plays critical roles in tissue protection, tissue repair and induction of antimicrobial peptides, while IL-17A amplifies immune responses by inducing IL-6 production, recruits monocytes and neutrophils by increasing local chemokine production, and modulates the pathogenesis of several autoimmune diseases by triggering overreacted inflammatory responses (15). IL-17A is also associated with exacerbated influenza-associated pathology (6, 49). Accumulated experimental and clinical evidence has broadened our understanding of the role of IL-17A in influenza virus infections and suggests that IL-17A-targeted immunotherapy may be a promising therapeutic option, whereas positive data on the therapeutic effect of antibodies targeting IL-17A on lung injury caused by influenza virus are still limited. An important reason is that although the IL-17A antibody can reduce the excessive activation of inflammation mediated by IL-17A, its reparative effect on damaged tissue needs to be improved. IL-22 is expected to improve this limitation of the IL-17A antibody. IL-22 is required for lung defense and repair after infection with influenza virus, as IL-22^−/−^ mice have more severe injury after virus infection (17, 50–53).

In this study, IL-22 was fused to the C-terminus of an anti-IL-17A antibody to endow the antibody with a tissue recovery function. The fusion protein retained the biological activity of the antibody and IL22 (**Figure 3**) and exhibited favorable stability *in vitro* (**Figure 2** and **Table 1**). The therapeutic efficacy of vmab-mIL22 was evaluated in mice with influenza A virus (H1N1)-induced lung immune injury. Treatment with vmab-mIL22 was more effective in depressing the pulmonary index and reducing pulmonary pathological damage than treatment with either vmab or IL22Fc alone (**Figure 4**). Our data demonstrated that vmab-mIL22, which can independently block the IL-17A pathway and activate the IL-22 pathway at the same time as a single reagent, had potent synergistic anti-inflammatory and repair-promoting activities in this model. This would also be an advantage of the fusion protein for the treatment of other acute and chronic inflammatory diseases. However, the fusion protein has larger molecular weight and more complex structures, which may be an adverse factor in the treatment of chronic inflammatory diseases. Because larger and more complex structures are more likely to produce anti-drug antibodies (ADA), which may weaken their therapeutic effect.

In the treatment of acute inflammatory diseases caused by influenza virus, we not only found that the fusion protein was better than the single administration group, but interestingly, the efficacy of the fusion protein was more significant than the combined administration, which was beyond our expectation. Previous studies have shown that for some antibody-cytokine fusion proteins, the cytokine moiety overrides antibody-mediated targeting, localizing the fusion protein to cytokine receptor-expressing cells (54). Therefore, despite the lack of antibody-mediated targeting, vmab-mIL22 may be recruited by cytokine-mediated targeting at disease sites where IL-22 receptors are highly expressed. Our results had shown that viral infections upregulate the expression of IL-22 receptors in the lung tissue, which is consistent with the previous study (50). The evidence suggested that vmab-mIL22 fusion protein may be more effectively enriched in disease sites due to the upregulation of IL-22 receptors, and therefore will achieve enhanced synergistic effect of blocking the IL-17A pathway and activating the IL-22 pathway. This may be one reason for its superior efficacy compared to combination therapy. Many cytokines secreted by immune cells have autocrine or paracrine functions. Therefore, vmab-mIL22 may be enriched to the disease site by binding to IL-22 receptors, where the antibody neutralizes locally secreted IL-17A. This is a unique advantage of the fusion protein and may partially explain why vmab-mIL22 exhibited better efficacy than the combination of vunakizumab and IL22.

In view of the excessive inflammation and injury in the lungs of severe patients infected with influenza virus, effective treatment strategies should focus on reducing the viral burden and inflammation, and promoting lung/epithelial repair. The combination of antiviral drugs and immunomodulators will be beneficial in the treatment of severe influenza. This requirement can be supported by the immunocytokine vmab-mIL22, which can independently target two separate pathways with a single reagent.

## Data Availability Statement

The datasets presented in this study can be found in online repositories. The names of the repository/repositories and accession number(s) can be found in the article/supplementary material.

## Ethics Statement

The animal study was reviewed and approved by the Animal Ethics Committee of the School of Pharmacy, Fudan University.

## Author Contributions

LH, CS, and XZ contributed equally to this work. All authors contributed to the article and approved the submitted version.

## Funding

This study was supported by grants from the National Natural Science Foundation of China (Grant Nos. 81773620, 82073752, 32070935,81673713 and 32000479) and the Shanghai Science and Technology Fund (20JC1411000 and 20S11904700).

## Conflict of Interest

The authors declare that the research was conducted in the absence of any commercial or financial relationships that could be construed as a potential conflict of interest.

## Publisher’s Note

All claims expressed in this article are solely those of the authors and do not necessarily represent those of their affiliated organizations, or those of the publisher, the editors and the reviewers. Any product that may be evaluated in this article, or claim that may be made by its manufacturer, is not guaranteed or endorsed by the publisher.
